# Decreasing dorsal cochlear nucleus activity ameliorates noise-induced tinnitus perception in mice

**DOI:** 10.1186/s12915-022-01288-1

**Published:** 2022-05-12

**Authors:** Thawann Malfatti, Barbara Ciralli, Markus M. Hilscher, Richardson N. Leao, Katarina E. Leao

**Affiliations:** 1 Hearing and Neuronal activity Lab, Brain Institute, Federal University of Rio Grande do Norte, Natal, Brazil; 2 Institute for Analysis and Scientific Computing, Vienna University of Technology, Vienna, Austria

**Keywords:** Tinnitus, Dorsal cochlear nucleus, Chemogenetics, Unit recording, GPIAS

## Abstract

**Background:**

The dorsal cochlear nucleus (DCN) is a region known to integrate somatosensory and auditory inputs and is identified as a potential key structure in the generation of phantom sound perception, especially noise-induced tinnitus. Yet, how altered homeostatic plasticity of the DCN induces and maintains the sensation of tinnitus is not clear. Here, we chemogenetically decrease activity of a subgroup of DCN neurons, Ca^2+^/Calmodulin kinase 2 *α* (CaMKII *α*)-positive DCN neurons, using Gi-coupled human M4 Designer Receptors Exclusively Activated by Designer Drugs (hM4Di DREADDs), to investigate their role in noise-induced tinnitus.

**Results:**

Mice were exposed to loud noise (9–11kHz, 90dBSPL, 1h, followed by 2h of silence), and auditory brainstem responses (ABRs) and gap prepulse inhibition of acoustic startle (GPIAS) were recorded 2 days before and 2 weeks after noise exposure to identify animals with a significantly decreased inhibition of startle, indicating tinnitus but without permanent hearing loss. Neuronal activity of CaMKII *α*+ neurons expressing hM4Di in the DCN was lowered by administration of clozapine-N-oxide (CNO). We found that acutely decreasing firing rate of CaMKII *α*+ DCN units decrease tinnitus-like responses (*p* = 3e −3, *n* = 11 mice), compared to the control group that showed no improvement in GPIAS (control virus; CaMKII *α*-YFP + CNO, *p* = 0.696, *n* = 7 mice). Extracellular recordings confirmed CNO to decrease unit firing frequency of CaMKII *α*-hM4Di+ mice and alter best frequency and tuning width of response to sound. However, these effects were not seen if CNO had been previously administered during the noise exposure (*n* = 6 experimental and 6 control mice).

**Conclusion:**

We found that lowering DCN activity in mice displaying tinnitus-related behavior reduces tinnitus, but lowering DCN activity during noise exposure does not prevent noise-induced tinnitus. Our results suggest that CaMKII *α*-positive cells in the DCN are not crucial for tinnitus induction but play a significant role in maintaining tinnitus perception in mice.

**Supplementary Information:**

The online version contains supplementary material available at (10.1186/s12915-022-01288-1).

## Background

Noise-induced tinnitus, commonly known as “ringing in the ears”, affects 10–15% of the world population [1, 2], where 1–2% seek medical assistance for severely decreased quality of life due to chronic tinnitus-related irritability, stress, anxiety, and/or depression [3–5]. The origin of tinnitus pathophysiology has been linked to the dorsal cochlear nucleus (DCN) of the auditory brainstem [5–9]; however, tinnitus generation and perception mechanisms are not well separated and far from completely understood.

Noise overexposure is known to alter firing properties of DCN cells [10–14], even after brief sound exposure at loud intensities [15]. Such alterations within the DCN circuits could relay abnormal signaling to higher auditory areas and confound spontaneous firing with sensory evoked input, generating tinnitus. It has been suggested that noise-induced tinnitus is partly due to an imbalance of excitation and inhibition within the DCN [5, 16] due to decrease in GABAergic [17] and glycinergic activity [18] for example. On the contrary, excitatory fusiform cells have been shown to increase burst activity [12, 19] following noise overexposure. Furthermore, a shift in bimodal excitatory drive of the DCN after noise overexposure have been shown due to down-regulation of vesicular glutamate transport 1 (VGlut1; auditory-related) and up-regulation of VGlut2 (somatosensory related) proteins in the cochlear nucleus [20, 21]. We have recently shown that directly manipulating the activity of Ca^2+^/Calmodulin kinase 2 *α* (CaMKII *α*)-positive DCN neurons in vivo using optogenetics can have distinct effects on unit activity of the DCN, also in neurons not responding directly to neither sound nor optogenetic light stimuli [22], highlighting how heavily interconnected the DCN circuit is [23]. DCN circuit disruption such as bilateral electrolytic DCN lesioning in rats has shown to prevent tinnitus generation [24]. Also, electrical stimulation of the DCN of rats can suppress tinnitus [25], and electrical high-frequency stimulation of the DCN with noise-induced tinnitus has shown to decrease tinnitus perception during tests [26]. This indicates that unspecific alterations of DCN activity can decrease tinnitus induction and perception, but if the same DCN populations are involved in the two mechanisms remains to be investigated.

Here, we behaviorally examine if tinnitus perception can be reduced by lowering the activity of CaMKII *α*-positive DCN neurons using chemogenetic hM4Di receptors, a Gi-coupled designer receptor that when activated induces membrane hyperpolarization and neuronal silencing [27] and suppression of synaptic transmission [28]. We have recently shown the CaMKII *α* promoter to be expressed by both excitatory and inhibitory DCN neurons, but with a preference for slow-firing units [22], presumable excitatory fusiform cells [29, 30]. We specifically investigated if noise-induced tinnitus can be ameliorated by lowering DCN neuronal activity. Next, we decrease CaMKII *α*+ DCN cell activity already during noise overexposure, to investigate how DCN circuit activity contributes to induction and maintenance of noise-induced tinnitus.

## Results

### Inhibition of CaMKII *α*-hM4Di-positive DCN cells decreases tinnitus perception

**Fig. 1.**
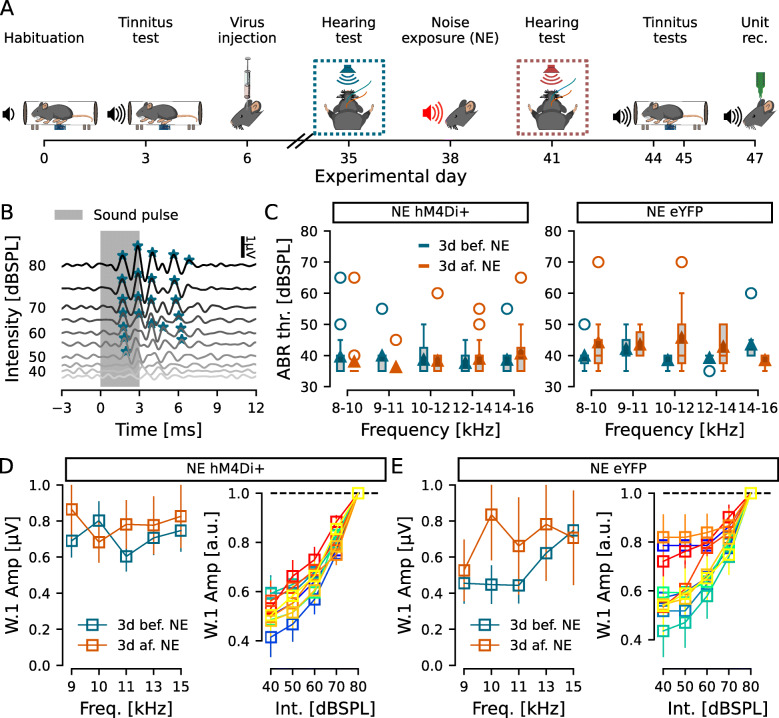
Noise exposure did not induce hearing threshold shifts. **A** Experimental timeline highlighting the ABR recordings before and after noise exposure. **B** ABR representative example for 8–10-kHz frequency presented from 80 to 35dBSPL. Algorith-detected response peaks are marked with asterisks. Hearing threshold was defined at the last intensity with an identified peak, in this example, 50dBSPL. **C** Group hearing thresholds for each frequency tested before (blue) and after (orange) noise exposure (NE) for hM4Di (left) and eYFP (right) injected animals. **D**, **E** ABR Wave 1 (W.1) amplitudes at 80dBSPL (left) and growth functions (right) for the hM4Di (**D**) and eYFP (**E**) injected animals. Left, amplitude of wave 1 for each frequency tested 3d before (blue) and 3d after (orange) nose exposure. Right, wave 1 amplitude at each tested intensity, normalized by amplitude at 80dBSPL, at each tested frequency (cold blue-green colors: 3d before; warm red-yellow colors: 3d after noise exposure). *n* = 11 and 7 for NE hM4Di+ and NE eYFP, respectively

**Fig. 2.**
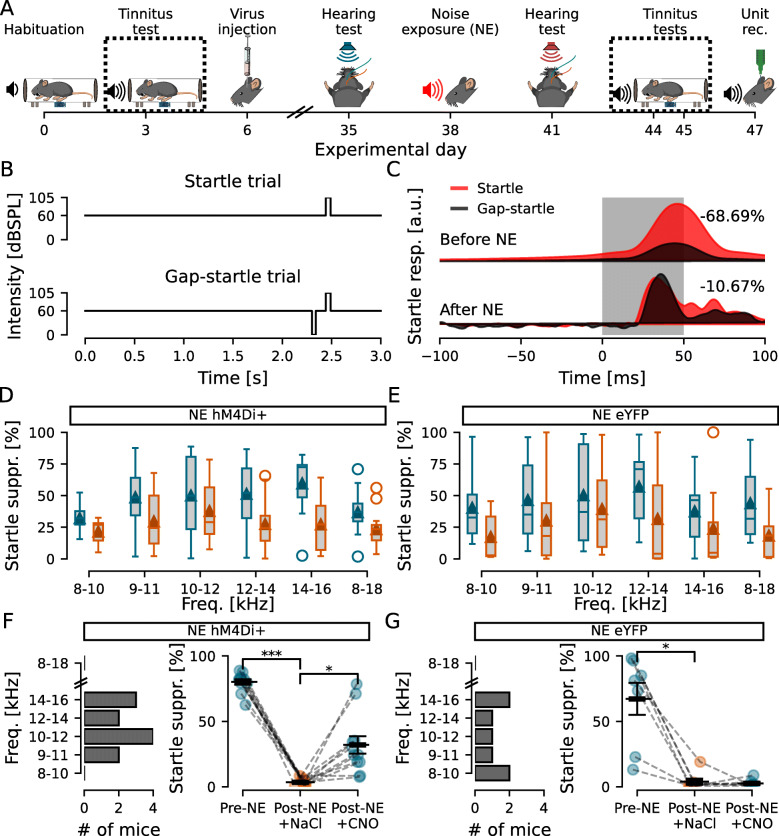
Inhibition of DCN CaMKII *α*-hM4Di-positive cells after noise exposure decreases tinnitus-like behavior. **A** Experimental timeline highlighting the GPIAS recordings before and after noise exposure. **B** Schematic drawing of the Startle and Gap-startle protocols. **C** Representative GPIAS recording of a mouse showing 68.69% suppression of acoustic startle before and 10.67% suppression after noise exposure when comparing Startle (red) and Gap-startle (black) responses, indicating tinnitus-like behavior for the tested frequency (9–11kHz). **D**, **E** GPIAS group performance before (blue) and after (orange) noise exposure for hM4Di (**D**) and eYFP (**E**) injected mice. **F**, **G** Left, histogram showing the number of hM4Di (**F**) and eYFP (**G**) injected animals in function of the frequency with the greatest decrease in GPIAS index. Right, GPIAS group index showed that hM4Di+ mice (**F**) decreased startle suppression after noise exposure and regained startle suppression under the effect of CNO, while eYFP-injected mice shows that, although presenting tinnitus-like responses after noise exposure, no difference was observed between NaCl and CNO treatments (*p* = 0.696). *n* = 11 and 7 for NE hM4Di+ and NE eYFP, respectively; *p >0.05; **p = 1.3e −10

To investigate perception of noise-induced tinnitus in mice using operant tasks can be challenging and has led to the development of a modified startle suppression task for rodents such as mice and guinea pigs [31–35]. Here, we initially screened for capability to carry out the gap prepulse inhibition of acoustic startle test (GPIAS [31, 36]). Mice were acclimatized and habituated to the test equipment before subjected to GPIAS (Fig. 1A) testing the capability of detecting a short (40ms) silence in background noise (60dBSPL at first, and 10dBSPL above individual hearing threshold after noise exposure) 100ms prior to a loud startle pulse (105dBSPL, 50ms duration, Fig. 2B), thereby suppressing the acoustic startle reflex by at least 30% [13]. Six different frequency bands were pseudo-randomly presented with the startle pulse (Startle session) or the silence in noise (Gap-startle session) and the startle suppression index was calculated for each frequency. Mice not showing gap detection capabilities for at least two frequencies were excluded from further experiments (4/34 mice, 11.8%; [13]).

Next, mice were injected bilaterally with viral vectors to transduce expression of inhibitory Designer Receptors Exclusively Activated by Designer Drugs (DREADDs; [27]) based on mutated muscarinic (M4) receptors (rAAV5/CaMKII *α*-HA-hM4D(Gi)-IRES-mCitrine, or for control experiments only a fluorescent protein, rAAV5/CaMKII *α*-eYFP) containing the CaMKII *α* promoter, in the DCN. Mice were returned to their home cage and left approximately 1 month (Fig. 1A) for adequate hM4Di expression in CaMKII *α* expressing cells, comprising both excitatory and some inhibitory cell populations [22]. Hearing threshold was evaluated by recording auditory brainstem responses (ABRs, Fig. 1) 3 days prior to noise exposure (1h, 90dBSPL, 9–11kHz filtered uniform white noise, followed by 2h in silence) under anesthesia in order to induce tinnitus-like behavior [37]. Recording of ABRs was repeated 3 days after noise exposure to examine any potential hearing threshold shift (Fig. 1A; [37]), as the aim was to study tinnitus mechanisms unrelated to persistent hearing loss. ABRs showed no significant difference in hearing threshold (hM4Di noise exposed: 41 ±0.9dBSPL, *n* = 11 mice; eYFP noise exposed: 47 ±1.1dBSPL; *n* = 7 mice) with no effect of exposure or frequency neither for the hM4Di group (eff. size of 3.9e −3 and 1.5e −2, *p* = 0.692 and 0.944, respectively; Fig. 1C left) nor the eYFP group (eff. size of 6.5e −3 and 5.7e −3, *p* = 0.847 and 0.53, respectively; Fig. 1C right). Also, no significant difference was found for any of the frequencies tested (Wilcoxon test, eff. size <0.34, *p* >0.1 for all frequencies; Fig. 1C). We also evaluated the amplitude of the first ABR wave at 80dBSPL to investigate if hair cell synapses or transmission of response through the auditory nerve were affected after the noise exposure; however, we found no significant effect of noise exposure, frequency, or interaction in the wave 1 amplitudes for the hM4Di group (*n* = 11, *F* <0.125, *p* >0.737; Fig. 1D left) or the eYFP injected group (*n* = 7, *F* <0.793, *p* >0.128; Fig. 1E left), similar to reported for noise-induced tinnitus in guinea pigs [20]. Similarly, the ABR growth functions showed only an effect of stimulus intensity (as expected, since the response amplitude is directly linked to the stimulus amplitude) for both the hM4Di group (*n* = 11, *F* = 70.602, *p* = 5.5e −17; Fig. 1D right) and the eYFP group (*n* = 7, *F* = 39.574, *p* = 1.2e −9; Fig. 1E right), and no effect of frequency, noise exposure, or any interaction for neither group (*F* <0.765, *p* >0.112; Fig. 1D, E right).

Next tinnitus-like perception was tested using GPIAS (Fig. 2A), with test rationale that if the animal has noise-induced tinnitus the animal will fail to perceive the silent gap (at a particular frequency), and thereby show lower gap-induced suppression of startle (Fig. 2B, C). When measuring the GPIAS response after noise exposure, mice received an i.p. injection of NaCl (same volume as for CNO treatment, 10 *μ*l/g), 30 min before the test, to perform the same procedures as for when subsequently activating inhibitory DREADDs. Group data of GPIAS indices did not reveal a significant effect of noise exposure, frequency, or interaction for the noise-exposed hM4Di+ group (*n* = 11; *F* = 2.1, 6.2, and 0.7; *p* = 0.081, 0.062, and 0.625, respectively; Fig. 2D) neither for the noise-exposed eYFP+ group (*n* = 7; *F* = 0.7, 6.2, and 0.9; *p* = 0.648, 0.088, 0.494, respectively; Fig. 2E). Pairwise comparisons indicate that noise exposure at 9–11kHz did not generate GPIAS deficits at one particular frequency tested (Fig. 2D, E, *n* = 11 and 7 mice, *p* >0.09 for all frequencies). Therefore, we report the most affected frequency band, defined as the frequency with the greatest index shift from before to after noise exposure (Fig. 2F, G left), as parameter for tinnitus [37]. Thereby, our model for noise-induced tinnitus showed that loud noise exposure could induce tinnitus-like responses in mice in both the noise-exposed hM4Di+ group (NE — initial startle suppression: 80.2 ±2.3%; post-NE with NaCl injection: 3.1 ±0.7%; *n* = 11 mice, *F* = 707.7, *p* = 1.3e −10; Fig. 2F right) and the noise-exposed eYFP+ group (initial startle suppression: 67.2 ±12.2%; post-NE with NaCl injection: 3.9 ±2.4%; *n* = 7, *F* = 24.2, *p* = 3e −3; Fig. 2G right) without a permanent hearing threshold shift. We evaluated whether the ABR shift was correlated with the GPIAS index shift at the GPIAS most affected frequency, but we found no significant correlation for neither the experimental group (NE hM4Di+; *n* = 11; *r* = 0.435, *p* = 0.18) nor the control group (NE hM4Di+; *n* = 7; *r* = 0.498, *p* = 0.255).

Next, we investigated if chemogenetically decreasing neuronal activity of the DCN can temporarily reduce tinnitus perception. For this, mice bilaterally expressing hM4Di DREADDs, or YFP for the control group, received an i.p injection of low-dose CNO (0.5mg/kg) 30 min prior [38] to the repeated GPIAS test session (Fig. 2A). Mice expressing hM4Di in the DCN that had showed tinnitus responses after noise exposure showed a significant improvement in detecting the silent gap under the effect of CNO compared to NaCl (31.8 ±7.3%; *n* = 11, *F* = 15.2, *p* = 3e −3; Fig. 2F right). Some mice showed drastic improvement as seen by the two outliers, while others showed more moderate improvement. However, removing those outliers from the dataset did not change the conclusion (post-NE with NaCl injection: 3.5 ±0.8; post-NE with CNO injection: 21 ±3.2; *n* = 9; *F* = 25.9, *p* = 9.4e −4). The control group, expressing eYFP, also with tinnitus responses after noise exposure showed no improvement after CNO injection compared to NaCl (2.6 ±1%; *n* = 7; *F* = 0.16, *p* = 0.696; Fig. 2G right). This indicates that lowering the activity of CaMKII *α*-hM4Di-positive cells in the DCN can acutely and partially ameliorate tinnitus.

### Unit recordings confirm hM4Di expressing cells chemogenetically decrease firing

**Fig. 3.**
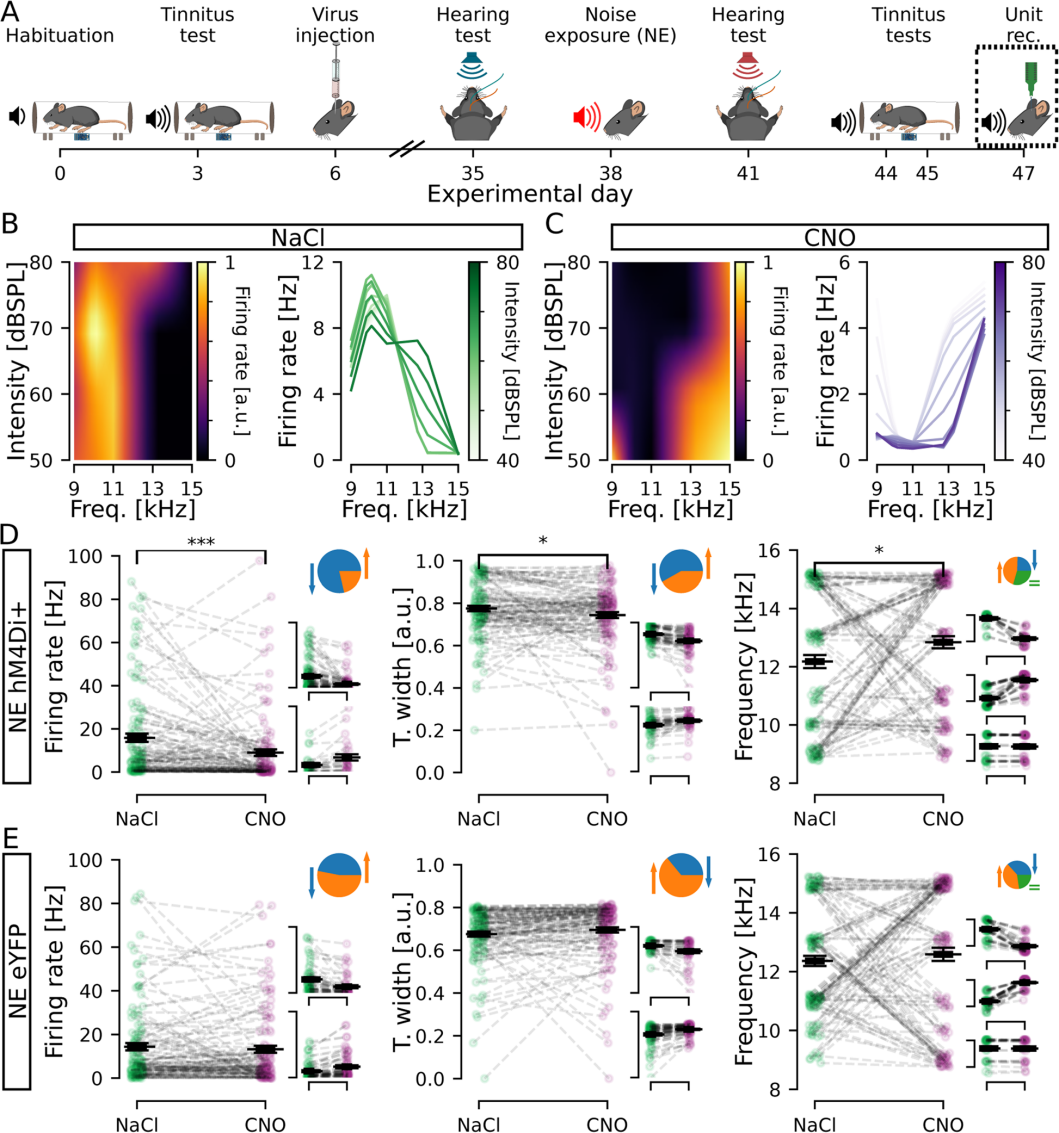
Decreasing CaMKII *α*-hM4Di-positive cell activity in the DCN changes firing properties of the circuitry. **A** Timeline of experiments highlighting unit recordings. **B**, **C** Left, normalized firing rate (colormap) of a representative unit after NaCl (**B**) and CNO (**C**) administration for each intensity (lines) and each frequency (columns) tested. Right, a different representation of the same representative examples in the left, showing firing rate per frequency for each intensity. Data was upsampled 3 times in the intensity and frequency dimensions. **D**, **E** Units firing rate (left), tuning width (middle), and best frequency (right) for stimulation at 80dBSPL, at each unit best frequency. Animals expressing hM4Di (**D**) showed a significant decrease in firing rate (left), decrease in tuning width (middle), and increase in best frequency (right). Control animals expressing eYFP (**E**) showed no significant change in any of those parameters. Individual unit values are shown in green (NaCl) or purple (CNO) condition. Black line indicates the mean ± SEM. Insets **D** and **E** (pie) shows the portion of units decreasing (blue), increasing (orange), or not changing (green) values upon CNO administration. Insets **D** and **E** (scatters) show distribution of unit values divided in groups for decrease, increase, or no change (for larger representation see Additional file 1: Fig. S1). *n* = 11 and 7 mice, 122 and 102 units, for NE hM4Di+ and NE eYFP, respectively; *p <0.05; ***p = 1.3e −4

We next wanted to understand how lowering the activity of CaMKII *α*-hM4Di-positive cells affected the whole DCN circuitry, both CaMKII *α*-positive and CaMKII *α*-negative units, and therefore performed in vivo unit recordings in the presence of CNO. Recent work has shown CNO to not pass the blood-brain barrier [39], instead reverting back to clozapine [40] at a ratio of clozapine to CNO of 7.4% in mice 30 min post-CNO injection [41], but with the ability to activate DREADD receptors at very low concentrations and avoiding off target effects [42]. Therefore, it was also important to directly confirm that CNO injections decreased DCN neuronal activity. DCN unit activity was recorded in response to short sound pulses (3ms; 8–10, 9–11, 10–12, 12–14, and 14–16kHz filtered uniform white noise) at different sound intensities (80–35dBSPL, 5dBSPL decreasing steps; presented at 10Hz). Spontaneous (5min) and sound-evoked activity was recorded using a 16-channel single-shank silicon probe lowered into the left DCN [22] in response to auditory stimuli following NaCl and CNO i.p. injections (30 min prior to recordings, Fig. 3A). A total of 122 and 102 units were isolated from 11 and 7 noise-exposed mice injected with CaMKII *α*-hM4Di and CaMKII *α*-eYFP, respectively. Units were analyzed for firing rate and best frequency (frequency eliciting the maximum firing rate within the 8–16kHz range tested) in response to sound stimuli (Fig. 3B, C, see Table 1). Administration of CNO significantly decreased the average firing rate in hM4Di expressing animals in response to 80dBSPL at best frequency (NaCl: 15.85 ±1.95Hz vs. CNO: 8.96 ±1.53Hz, *p* = 1.3e −4, Fig. 3D left). Examining units from hM4Di+ mice in detail showed that the majority of units (96/122) decreased firing rate (66 ±2% decrease in firing frequency; Fig. 3D left insets; Additional file 1: Fig. S1A, middle) and 26/122 units increased firing rate following CNO administration (132 ±28% increase; Fig. 3D left insets; Additional file 1: Fig. S1A, right). In control animals expressing eYFP, CNO injections did not significantly change the average firing rate of units (NaCl: 14.36 ±1.67Hz vs. CNO: 13.21 ±1.62Hz, *n* = 102 units from 7 mice, *p* = 0.4, Fig. 3E left). As auditory neurons are developmentally tuned to respond better to certain frequencies, we further analyzed tuning width and any change in best frequency of each unit within the 8–16kHz range tested. For tuning width, lower values represent narrower frequency response peaks. Here, we found an average decrease in tuning width following CNO administration (0.78 ±0.01 to 0.74 ±0.01, *p* = 1.9e −2, Fig. 3D middle), but after closer examination 71/122 (58%) units decreased while 51/122 (42%) increased tuning width in response to the short sound pulses tested (Fig. 3D middle insets; Additional file 1: Fig. S1B). No significant changes were observed in control eYFP animals (0.67 ±0.01 to 0.69 ±0.01, *p* = 0.094, Fig. 3E middle). Finally, we tested if units changed to what frequency they display maximum firing rate (best frequency) after CNO injection. Data showed a small but significant average increase in best frequency (12.16 ±0.22Hz to 12.83 ±0.21Hz, *p* = 2.6e −2, Fig. 3D right), with 57/122 (47%) increasing, 30/122 (24%) decreasing, and 35/122 units (29%) maintaining the same best frequency for both treatments (Fig. 3D right insets; Additional file 1: Fig. S1C). Taken together, electrophysiological data shows that inhibition of CaMKII *α*-hM4Di-positive DCN cells indeed lowers the average firing rate of DCN neurons, as well as affecting tuning width and best frequency in the DCN circuitry within the 8–16kHz range, which may decrease the tinnitus perception as seen by behavioral improvement of GPIAS after CNO administration.

**Table 1:** Firing rate, tuning width, and best frequency features for each experimental group (NE hM4Di+ — animals exposed to noise expressing CaMKII *α*-hM4Di, *n*=11 mice; or NE+CNO hM4Di — animals exposed to noise under effect of CNO, expressing CaMKII *α*-hM4Di, *n*=6 mice) and each respective control (NE eYFP — animals exposed to noise expressing CaMKII *α*-eYFP, *n*=7 mice; or NE+CNO eYFP — animals exposed to noise under effect of CNO, expressing eYFP, *n*=6 mice) represented as mean ± standard error of the mean (SEM). Unit responses are further subdivided based on the applied treatment (NaCl or CNO) and on the CNO response in relation to NaCl (All — all units; Decreased and Increased — units that show a decrease or an increase in that feature under effect of CNO, respectively). All *p* values were Bonferroni-corrected for multiple comparisons

	All		Decreased		Increased	
	NaCl	CNO	NaCl	CNO	NaCl	CNO
Firing rate (Hz; mean ± SEM)						
NE hM4Di+	15.85 ±1.95	8.96 ±1.53	17.45 ±2.32	5.57 ±1.22	9.92 ±2.92	21.52 ±4.82
NE eYFP	14.36 ±1.67	13.21 ±1.62	20.35 ±3.04	9.23 ±2.2	9.61 ±1.55	16.38 ±2.25
NE+CNO hM4Di+	9.37 ±0.67	8.45 ±0.6	9.9 ±0.92	7.06 ±0.69	8.43 ±0.88	10.88 ±1.08
NE+CNO eYFP	4.81 ±0.68	4.24 ±0.59	5.77 ±1.09	3.23 ±0.59	4.04 ±0.81	5.9 ±1.14
Tuning width (a.u.; mean ± SEM)						
NE hM4Di+	0.78 ±0.01	0.74 ±0.01	0.82 ±0.01	0.72 ±0.02	0.71 ±0.02	0.78 ±0.02
NE eYFP	0.67 ±0.01	0.69 ±0.01	0.71 ±0.01	0.62 ±0.03	0.65 ±0.02	0.73 ±0.01
NE+CNO hM4Di+	0.61 ±0.01	0.63 ±0.01	0.59 ±0.02	0.53 ±0.02	0.62 ±0.01	0.69 ±0.01
NE+CNO eYFP	0.38 ±0.02	0.4 ±0.02	0.42 ±0.03	0.36 ±0.03	0.35 ±0.03	0.43 ±0.03
Best freq. (kHz; mean ± SEM)						
NE hM4Di+	12.16 ±0.22	12.83 ±0.21	14.4 ±0.23	10.53 ±0.25	10.54 ±0.24	14.07 ±0.21
NE eYFP	12.37 ±0.18	12.52 ±0.23	13.19 ±0.25	9.96 ±0.21	10.94 ±0.17	14.57 ±0.13
NE+CNO hM4Di+	12.15 ±0.15	11.7 ±0.15	12.86 ±0.2	10.0 ±0.1	10.52 ±0.2	13.22 ±0.24
NE+CNO eYFP	10.87 ±0.21	10.91 ±0.2	12.74 ±0.42	10.09 ±0.21	10.11 ±0.2	12.39 ±0.38

### Decreasing CaMKII *α*-hM4Di-positive DCN cell activity during noise exposure does not prevent tinnitus-like behavior

**Fig. 4.**
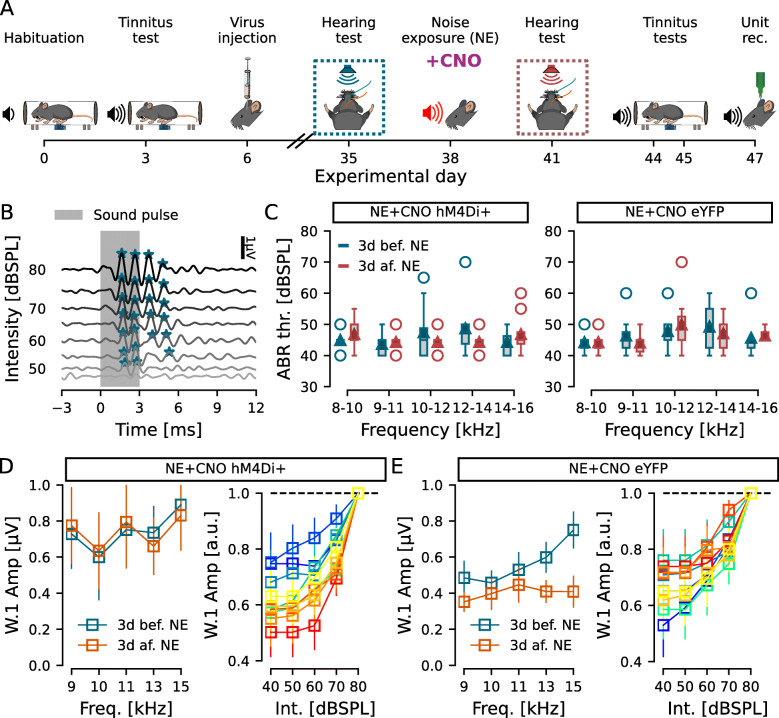
Inhibiting DCN CaMKII *α*-hM4Di-positive cell activity during noise exposure does not interfere with hearing at the tested frequencies. **A** Timeline of experiments highlighting ABR recordings before and after noise exposure. **B**, **C** Representative ABR traces (**B**) and group responses for hM4Di (**C** left) and eYFP (**C** right) injected mice that received i.p. CNO injection 30 min before noise exposure. **D**, **E** ABR Wave 1 (W.1) amplitudes at 80dBSPL (left) and growth functions (right) for the hM4Di (**D**) and eYFP (**E**) injected animals that received CNO before noise exposure. Left, amplitude of wave 1 for each frequency tested 3d before (blue) and 3d after (orange) noise exposure. Right, wave 1 amplitude at each tested intensity, normalized by amplitude at 80dBSPL, at each tested frequency (cold blue-green colors: 3d before; warm red-yellow colors: 3d after noise exposure). *n* = 6 and 6 for NE+CNO hM4Di+ and NE+CNO eYFP

In an attempt to investigate if CaMKII *α*-hM4Di-positive DCN cells are directly part of noise-induced tinnitus plasticity, we performed a new set of experiments where CNO was administered 30 min prior to noise exposure to obtain DREADD-mediated inhibition during noise exposure (Fig. 4A). ABRs before and after noise exposure confirmed no hearing threshold shift at the tested frequency bands (*n* = 6 hM4Di and 6 eYFP mice), with no effect of exposure or frequency (Kruskal-Wallis test, eff.size of 6.5e −3 and 5.7e −3, *p* = 0.847 and 0.53, respectively; Fig. 4B, C) and no significant differences at any frequency tested (Wilcoxon test, eff. size <0.4, *p* >0.17 for all frequencies; Fig. 4C). We again evaluated the amplitude of the ABR wave 1 at 80dBSPL and, similar to the previous dataset (Fig. 1), we found no significant effect of noise exposure, frequency, or interaction in the wave 1 amplitudes for the hM4Di group (*n* = 6, *F* <0.12, *p* >0.748; Fig. 4D left) or the eYFP injected group (*n* = 6, *F* <1.225, *p* >0.312; Fig. 4E left). Also, the ABR growth functions showed only an effect of stimulus intensity for both the hM4Di group (*n* = 6, *F* = 20.231, *p* = 8.4e −7; Fig. 4D right) and the eYFP group (*n* = 6, *F* = 11.682, *p* = 1.9e −4; Fig. 4E right) and no effect of frequency, noise exposure, or any interaction for neither group (*F* <0.704, *p* >0.512; Fig. 4D, E right).

**Fig. 5.**
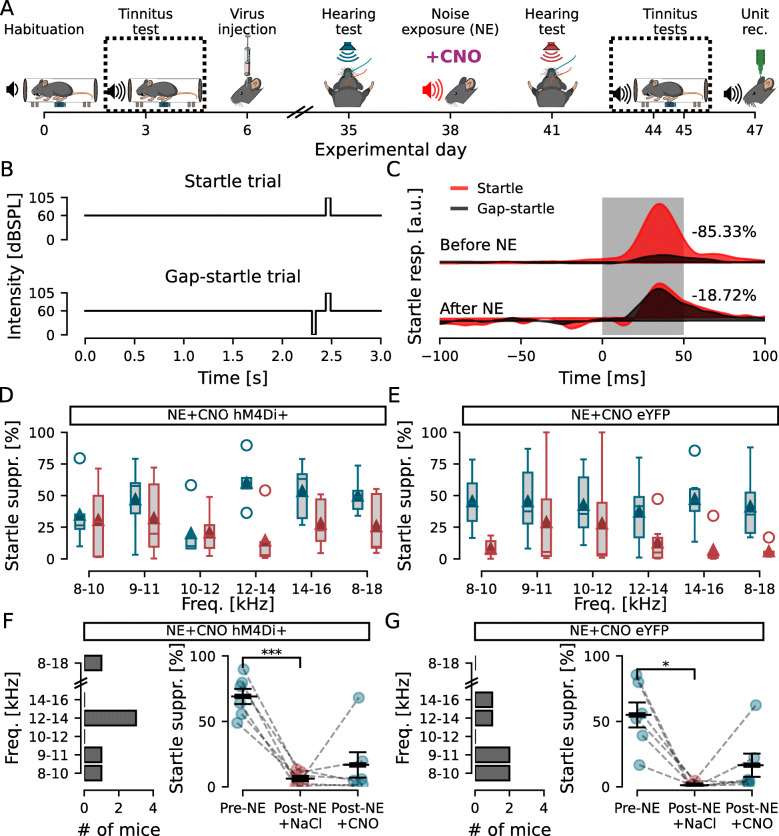
Decreasing CaMKII *α*-hM4Di-positive DCN cell activity during noise exposure does not prevent tinnitus-like behavior and abolishes hM4Di-dependent recovery. **A** Schematic timeline highlighting GPIAS recordings before and after noise exposure. **B** Schematic outline of Startle and Startle-gap protocols. **C** Representative GPIAS response. **D**, **E** Group results for startle suppression of all frequencies tested before (blue) and after (dark red) noise exposure in the presence of CNO for hM4Di (**D**) and eYFP (**E**) injected mice. **F**, **G** Left, quantification of most affected frequency of each animal. Right, Inhibition of CaMKII *α*-hM4Di-positive DCN cells during noise exposure did not prevent a decrease in the startle suppression, indicating tinnitus, and also the second CNO injection before GPIAS recording (in mice receiving CNO 30 min before the noise exposure) did not recover startle suppression (**F**; *p* = 0.404). The eYFP injected group showed tinnitus-like behavior after noise exposure with CNO and did not recover the startle suppression after CNO injection upon testing GPIAS (**G**; *p* = 0.176). *n* = 6 and 6 for NE+CNO hM4Di+ and NE+CNO eYFP; *p <0.05

Since, similarly for the previous dataset, we found no generalized effect of the noise exposure, frequency or interaction for neither noise-exposed+CNO hM4Di+ (*n* = 6; *F* = 0.7, 0.1, and 2.2; *p* = 0.617, 0.725, and 0.093, respectively; Fig. 5A–D) nor noise-exposed+CNO eYFP+ group (*n* = 6; *F* = 0.3, 4.4, and 1.2; *p* = 0.882, 0.104, and 0.33, respectively; Fig. 4E), we again grouped the most affected frequency band of each animal. We found that inhibition of CaMKII *α*-hM4Di+ DCN neurons during noise exposure did not prevent startle suppression deficit after noise exposure compared to the initial screening (*n* = 6; 67.5 ±6.8% pre-NE; 5.3 ±2.2% post-NE + NaCl; *F* = 114.2, *p* = 1.2e −4; Fig. 5F), indicating that lowering CaMKII *α*-hM4Di+ DCN cell activity could not prevent noise-induced tinnitus-like behavior. Furthermore, we found no significant correlation between the ABR shifts and GPIAS index shifts at the most affected frequency for neither the experimental group (NE+CNO hM4Di+; *n* = 6; *r* = 0.762, *p* = 0.078) nor the control group (NE+CNO hM4Di+; *n* = 6; *r* = −0.665, *p* = 0.15). This indicates that initial noise trauma may be more related to cochlear overexcitability and that lowering activity of the DCN during loud noise does not have any overall protective effect on noise-induced tinnitus.

Surprisingly, in this set of experiments (Fig. 5A), we found average GPIAS responses to not show any improvement in tinnitus-like responses when lowering activity of CaMKII *α*-hM4Di+ DCN cells that were inhibited during noise-exposure (*n* = 6 mice; 5.3 ±2.2% post-NE + NaCl; 16.2 ±11.6% post-NE + CNO; *F* = 0.8, *p* = 0.404; Fig. 5F). The control group, as expected, showed tinnitus-like responses after noise exposure and did not show any improvement in startle suppression after the CNO i.p. injection (*n* = 6; 54.9 ±9.6% pre-NE; 1.2 ±0.7% post-NE + NaCl; 16.5 ±8.8% post-NE + CNO; *F* = 25.7, *p* = 4e −3 for pre-NE vs. post=NE + NaCl; *F* = 2.5, *p* = 0.176 for post-NE + NaCl vs. post-NE + CNO; Fig. 5G). Together, these experiments suggest that lowering the activity of CaMKII *α*-hM4Di-positive DCN cells during noise exposure does not prevent tinnitus-like behavior, thereby CaMKII *α*+ DCN neuron activity does not appear crucial during noise exposure for triggering tinnitus.

### Lowered neuronal activity during noise exposure still renders units affected by CNO

**Fig. 6.**
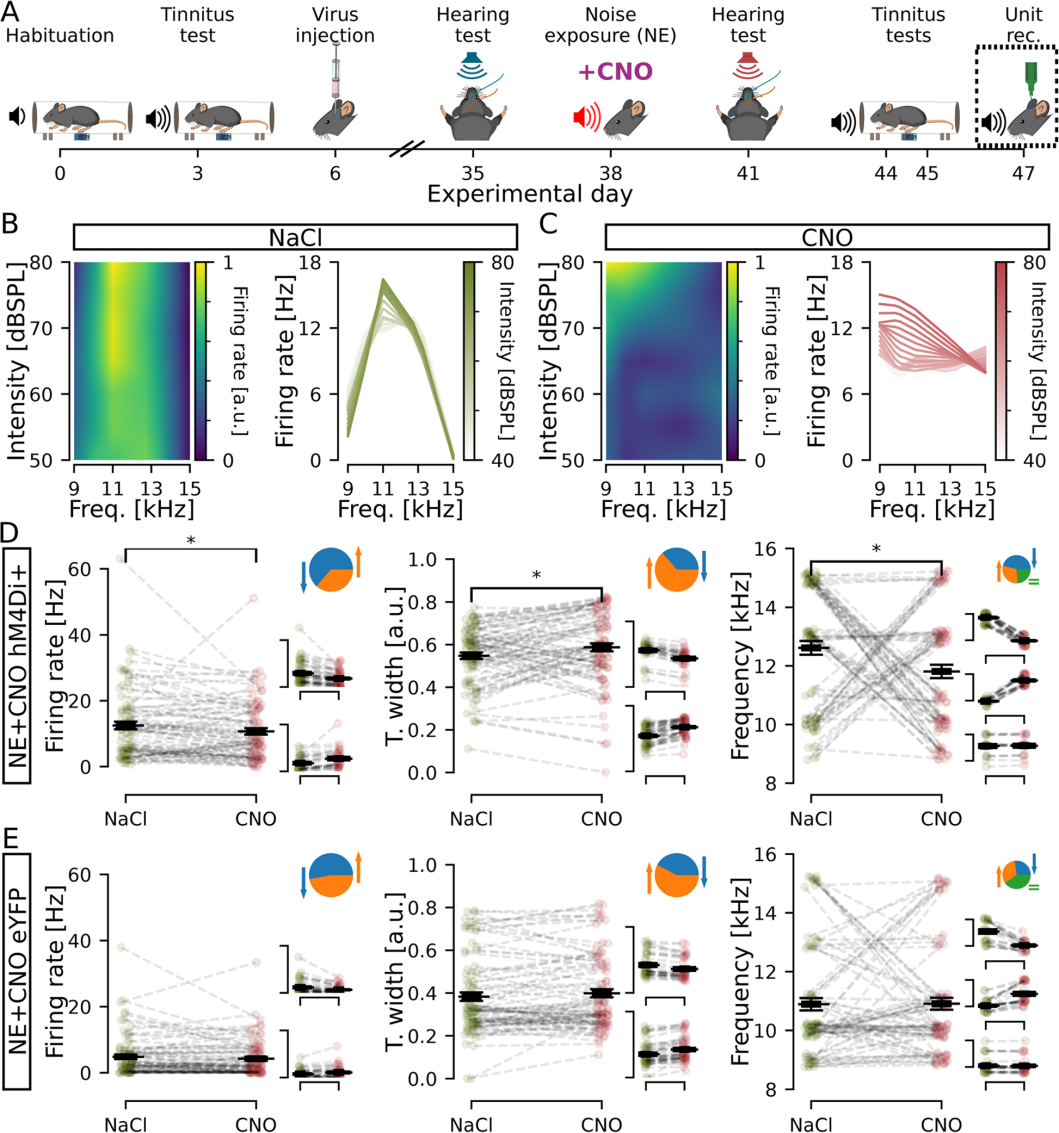
Decreasing activity of CaMKII *α*-hM4Di-positive DCN cells that were also inhibited during noise exposure changes firing properties of the circuitry. **A** Timeline of experiments highlighting the unit recordings. **B**–**C** Left, normalized firing rate (colormap) of a representative unit after NaCl (**B**) and CNO (**C**) injection for each intensity (lines) and each frequency (columns) tested. Right, a different representation of the same representative examples in the left, showing firing rate per frequency for each intensity. **D**, **E** Units firing rate (left), tuning width (middle), and best frequency (right) for stimulation at 80dBSPL, at each unit best frequency in the 8–16kHz interval. **D** Units from mice expressing hM4Di, showing significant difference after CNO application for firing rate, tuning width, and best frequency. Individual unit values are shown in olive (NaCl) or red (CNO) condition. Black line indicates the mean ± SEM. **E** Units from mice expressing eYFP showing no significant difference for any of the parameters. Insets show the proportion of units decreasing (blue), increasing (orange) or not changing (green) parameters of each graph (see Additional file 2: Fig. S2 for greater detail). *n* = 6 and 6 mice, 85 and 91 units, for NE+CNO hM4Di+ and NE+CNO eYFP; *p <0.05

To better understand the role of neural activity of the DCN during noise exposure, we investigated if CNO administration lowered CaMKII *α*-hM4Di-positive DCN unit activity in animals that also had received CNO *30 min prior* to the noise exposure (Fig. 6A). Again, we compared firing frequency, tuning width, and best frequency within the 8–16kHz range tested in the presence of NaCl or CNO (Fig. 6B, C, Table 1). We found that a CNO i.p. injection led to a significant decrease in firing rate (12.5 ±1.1Hz to 10.7 ±0.9Hz; *n* = 85 units from 6 mice; *p* = 4.6e −2; Fig. 6D left) in animals expressing hM4Di, but not in control animals (4.8 ±0.7Hz to 4.2 ±0.6Hz; *n* = 91 units from 6 mice; *p* = 0.195; Fig. 6E left). Also, average unit tuning width increased (0.548 ±0.01 to 0.587 ±0.01; *p* = 1.09e −2; Fig. 6D middle) and average best frequency decreased (12.6 ±0.2Hz to 11.8 ±0.2Hz; *p* = 4.9e −2; Fig. 6D right), while the control group, expressing only eYFP, showed no significant changes in either of the parameters (*p* = 0.104 and 0.113, respectively; Fig. 6E middle and right, Table 1). Although the average response showed a significant decrease in firing frequency upon CNO administration, the modulation appeared bidirectional with 54 unit decreasing and 31 units increasing firing rate (Fig. 6D left insets; Additional file 2: Fig. S2A). Similar results were seen for tuning width (31 units decreasing and 54 units increasing, Fig. 6D middle insets) and best frequency (39 units decreasing, 46 units increasing, Fig. 6D right insets, Additional file 2: Fig. S2B-C). Interestingly, the unit firing rate from animals pre-treated with CNO during noise exposure was mostly below 40kHz in these experiments, indicating a smaller sample of high-frequency firing units in these animals, or that typical fast spiking units fired at a lower frequency. Taken together, CNO administration still significantly decreased DCN unit activity, despite not showing improvement in GPIAS index. Interestingly, the unit recordings show, from both sets of experiments (without and with CNO before the noise exposure) that the reduction in average firing frequency for noise-exposed mice (16Hz decreasing to 9Hz) was less dramatic in mice injected with CNO before the noise exposure (9Hz decreasing to 8.5Hz, see Table 1). Therefore, a small average reduction in firing frequency is not sufficient for improving the GPIAS index.

### DCN units are differently modulated by DREADDs if activity was also lowered during the noise exposure

**Fig. 7.**
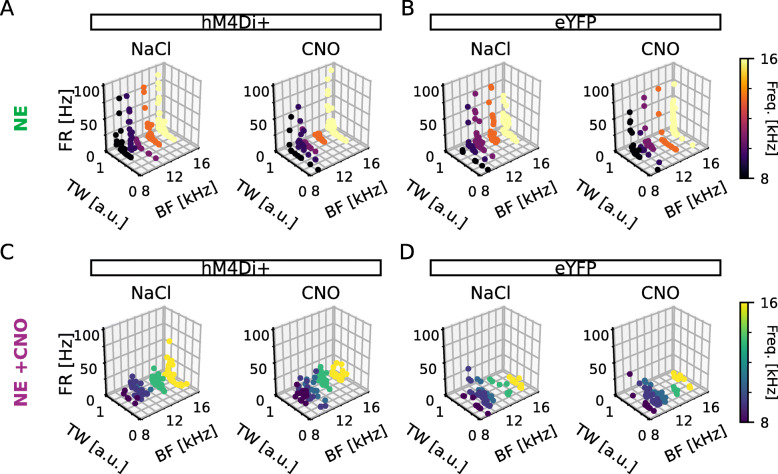
Three-dimensional scatter plots of firing rate, tuning width, and best frequency of DCN units of noise-exposed hM4Di+ or eYFP+ animals in the presence of NaCl or CNO. **A**, **B** 3D scatters representing each unit by *firing rate x tuning width x best frequency* for hM4Di (experimental; **A** and eYFP (control; **B** animals under NaCl (left) or CNO (right) treatment. **C**, **D** Same as **A** and **B** for experiments where animals were administered CNO (0.5mg/kg) 30 min prior to noise exposure. Colors represent the best frequency response between 8 and 16kHz. FR, firing rate; TW, tuning width; BF, best frequency; *n* = 11, 7, 6, and 6 mice; 122, 102, 85, and 91 units; for NE hM4Di+, NE eYFP, NE+CNO hM4Di+ and NE+CNO eYFP, respectively

To better understand alterations in firing patterns of the DCN when activating inhibitory DREADDs, we did correlation analysis of all obtained data. It is important to note that recordings were from any unit in the vicinity of the electrode (both CaMKII *α*+ and CaMKII *α*− units) responding to sound while CaMKII *α*+ neurons of the DCN were chemogenetically inhibited. Although CNO (0.5mg/kg) administration consistently lowered the average firing rate in animals expressing hM4Di in DCN CaMKII *α*+ neurons, the bidirectional modulation seen when looking at individual DCN unit responses to sound after CNO administration made us specifically question whether any correlation exist between firing rate, tuning width, and best frequency within the 8–16kHz range in response to CNO (Table 2). Here, we display the unit features as 3-dimensional plots for hM4Di+ and control animals (Fig. 7) that received CNO 30 min prior to the GPIAS test to ameliorate from tinnitus (Fig. 7A, B) and from hM4Di+ and control animals receiving CNO both 30 min prior to the noise exposure and the GPIAS test (Fig. 7C, D) and examined any correlation between unit parameters using Pearson correlation coefficient (*r*), with the *p*-value testing non-correlation (Table 2). We divided our analysis in comparing all units for each experimental group, but also subdivided analysis in units decreasing or increasing firing upon CNO administration, respectively (Table 2). In the first set up experiments, trying to recover tinnitus-like behavior (Figs. 1, 2, and 3), we found no correlation between average firing rate and best frequency for either experimental group, suggesting that decreasing CaMKII *α*-hM4Di+ cell firing rate does not alter units tuning to a certain frequency. Firing rate and tuning width appeared equally correlated in the presence of NaCl or CNO, indicating that lowering CaMKII *α*-hM4Di+ cells activity using DREADDs does not decouple the existing correlation between firing rate and tuning width. However, when splitting data into units either decreasing (96/122) or increasing (26/122) firing rate in response to CNO, it appears that units decreasing firing rate upon CNO administration no longer correlate with tuning width, meaning that units showing low firing rate do not necessarily have a low tuning width (Table 2; Additional file 1: Fig. S1A-B). In experiments where CNO was given during the noise exposure in an attempt to prevent tinnitus-like behavior (Figs. 4, 5, and 6), we instead noted that firing rate was not correlated with tuning width. Interestingly, CNO administration during unit recordings appeared to recover this missing correlation (Table 2). This could indicate that CNO during noise exposure can influence lateral inhibition within the DCN circuitry, since the firing rate is no longer coupled to the tuning of response to sound, for example units responding with a low firing rate but broadly to neighboring frequencies.

**Table 2:** Correlation pairs of firing rate (FR), tuning width, and best frequency features for each experimental group (NE hM4Di+ — animals exposed to noise expressing CaMKII *α*-hM4Di, *n*=11 mice; or NE+CNO hM4Di — animals exposed to noise under effect of CNO, expressing CaMKII *α*-hM4Di, *n*=6 mice) and each respective control (NE eYFP — animals exposed to noise expressing CaMKII *α*-eYFP, *n*=7 mice; or NE+CNO eYFP — animals exposed to noise under effect of CNO, expressing eYFP, *n*=6 mice) represented as Pearson correlation coefficient (*r*) and *p*-value for testing non-correlation (*p*). Unit responses are further subdivided based on the applied treatment (NaCl or CNO) and on the firing rate change under CNO in relation to NaCl treatment (All — all units; Decreased and Increased — units that show a decrease or an increase in firing rate under effect of CNO, respectively). All *p* values were Bonferroni-corrected for multiple comparisons

	All		Decreased FR after CNO		Increased FR after CNO	
	NaCl	CNO	NaCl	CNO	NaCl	CNO
Firing rate x Best freq. (r, p)						
NE hM4Di+	0.064, 1.000	0.059, 1.000	0.085, 1.000	-0.094, 1.000	-0.031, 1.000	0.047, 1.000
NE eYFP	0.164, 0.639	0.142, 1.000	0.119, 1.000	0.370, 0.053	0.067, 1.000	-0.081, 1.000
NE+CNO hM4Di+	-0.081, 1.000	-0.024, 1.000	0.022, 1.000	-0.061, 1.000	-0.323, 0.058	0.007, 1.000
NE+CNO eYFP	0.069, 1.000	0.023, 1.000	0.003, 1.000	0.229, 1.000	0.054, 1.000	-0.224, 1.000
Firing rate x Tuning width (r, p)						
NE hM4Di+	0.479, 2.2e-7*	0.352, 6.1e-4*	0.504, 1.5e-6*	0.264, 0.083	0.413, 0.324	0.484, 0.108
NE eYFP	0.378, 1.6e-4*	0.445, 2.5e-6*	0.557, 1.1e-4*	0.470, 3.0e-3*	0.349, 3.2e-2*	0.429, 2.3e-3*
NE+CNO hM4Di+	0.045, 1.000	0.246, 5.3e-3*	0.099, 1.000	0.293, 9.9e-3*	-0.109, 1.000	0.140, 1.000
NE+CNO eYFP	0.470, 5.0e-5*	0.481, 2.9e-5*	0.469, 1.1e-2*	0.390, 0.074	0.492, 2.4e-2*	0.596, 1.4e-3*
Tuning width X Best freq. (r, p)						
NE hM4Di+	0.301, 6.7e-3*	0.352, 6.3e-4*	0.201, 0.450	0.314, 1.6e-2*	0.591, 1.4e-2*	0.249, 1.000
NE eYFP	0.198, 0.252	0.252, 4.7e-2*	0.182, 1.000	0.138, 1.000	0.247, 0.378	0.263, 0.27
NE+CNO hM4Di+	0.069, 1.000	0.137, 0.522	0.064, 1.000	0.108, 1.000	0.086, 1.000	0.181, 1.000
NE+CNO eYFP	0.068, 1.000	0.231, 0.306	-0.110, 1.000	0.365, 0.126	0.223, 1.000	0.036, 1.000

**Fig. 8.**
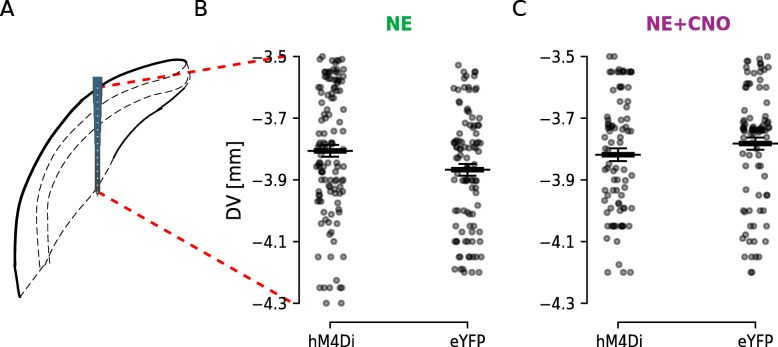
DCN unit depth profile. **A** Schematic representation of the probe location within the DCN according to coordinates used highlighting the dorsoventral depth of unit recordings. **B** Distribution of recorded DCN units along the dorsoventral axis for noise-exposed animals expressing CaMKII *α*-hM4Di or CaMKII *α*-eYFP. **C** The same as **B** but for experimental and control animals that were pre-treated with CNO 30 min before noise exposure. Black bars indicate mean ± SEM. *n* = 11, 7, 6, and 6 mice; 122, 102, 85, and 91 units; for NE hM4Di+, NE eYFP, NE+CNO hM4Di+, and NE+CNO eYFP, respectively

Interestingly, CNO administration prior to noise exposure also showed a particular loss of correlation between firing rate and tuning width in control animals, for units decreasing firing rate following CNO administration compared to NaCl (NaCl *r*,*p*: 0.469, 1.1e −2; CNO *r*,*p*: 0.39, 0.074). This suggests that CNO, converted to clozapine, could have small electrophysiological effects on the DCN circuitry that is not seen behaviorally nor in averaged data (Fig. 6, Table 2). When investigating correlations between tuning width and best frequency, we only observed correlations between the parameters in the groups with noise exposure without pharmacological manipulation. The correlation between tuning width and best frequency was seen for units decreasing firing rate upon CNO administration, but for units that increased firing frequency upon CNO administration this correlation was lost (NaCl *r*,*p*: 0.591, 1.4e −2; CNO *r*,*p*: 0.249, 1.0). We again observed a correlation between tuning width and best frequency in control animals only appearing following CNO administration. This correlation was however lost when units were divided into increasing or decreasing firing frequency following CNO administration. Still, it highlights the possibility that clozapine has small electrophysiological effects despite the very low-dose CNO used in this study [42, 43]. Finally, we did not record from units of either experimental group (noise-exposed or noise-exposed + CNO) at any particular depth or layer, as we did not want to bias data to any particular frequency region of the DCN (Fig. 8). In summary, these experiments show that decreasing the overall DCN firing rate in response to sound acutely can improve gap-startle suppression, indicating a reduction in tinnitus perception.

## Discussion

Here, we found that decreasing activity of CaMKII *α*-hM4Di-positive DCN cells after noise exposure can decrease tinnitus-like responses and showed that activity of the DCN is involved in the maintenance of tinnitus perception. Still, many higher areas are most likely also involved in tinnitus but this study shows that lowering neuronal activity of the dorsal cochlear nucleus might be able to counteract increased activity or gain observed in higher auditory areas after noise exposure [9, 44]. Moreover, this DCN subpopulation does not appear to have an important role in initial tinnitus generating mechanisms, since inhibiting CaMKII *α*-hM4Di-positive DCN cells during noise exposure did not prevent development of tinnitus-like response. Instead, decreasing firing capability of CaMKII *α*+ DCN neurons during noise exposure abolished CNO-dependent recovery after noise exposure. This suggests that CaMKII *α*-positive neurons play a role in tinnitus maladaptive plasticity but not during the temporal window of the noise exposure.

In this study, we aimed to not confound mechanisms of increased neuronal activity due to noise exposure with plasticity related to partial hearing loss as several studies in children, adolescence, and adults show the prevalence of noise-induced tinnitus with normal audiograms [45–47]. This is an important group to consider, especially since noise-induced tinnitus is becoming more common in youth [48–50]. In comparison to other animal models of noise-induced tinnitus, our noise exposure is on the lower edge of parameters often used [19–21, 26, 51–55]. We used 90dBSPL for 1h followed by 2h of silence, as we have previously shown to be able to generate tinnitus-like behavior without permanent threshold shifts [37]. Here, we show again that noise exposure at 9–11kHz does not cause altered GPIAS index at any particular frequency, similarly to studies of mice and guinea pigs [33, 56], as well as for patients reporting noise exposure as tinnitus etiology [57]. Unfortunately, due to a low signal-to-noise ratio (SNR) of the GPIAS recordings, it was not possible to analyze responses to single trials, which would enable the investigation of tinnitus-like responses in individual animals. Such analysis would be possible having better SNR conditions, such as using more sensitive vibration sensors or more stable platforms. Alternatively, different animal models might enable different measures such as the Preyer reflex instead of the startle response in guinea pigs, which can be more reliable and enable such single-animal analysis [58].

It is well known that the DCN circuitry presents altered firing following noise exposure [9]. DCN cells, specially fusiform cells, can increase spontaneous activity [8], bursting activity, and synchrony [19]. The mechanisms behind altered DCN plasticity during a loud noise exposure are still not completely understood, but bimodal stimulation experiments in salicylate-treated guinea pigs show increased firing rate (indicative of long-term potentiation) in fusiform cells within GPIAS-confirmed tinnitus bands and indications of long-term depression of fusiform cells outside the tinnitus frequency band [59]. Here, we tried to counteract an increased firing rate during GPIAS tests and unit recordings using chemogenetics, still acknowledging that clozapine-N-oxide administration is not temporally precise. For example, it has been shown that CNO has a half-life of 2h in mice, and with biological effects lasting 6–10h [38]. Here, we saw that decreasing the activity of CaMKII *α*-hM4Di-positive DCN subpopulation, during the loud noise exposure, could not counteract activity of the auditory system enough to prevent tinnitus in mice. How long the CNO effects persist and potential downstream targets were not assessed in this study, and additional studies with repeated CNO administration over longer periods following tinnitus induction would be interesting to evaluate.

**Fig. 9.**
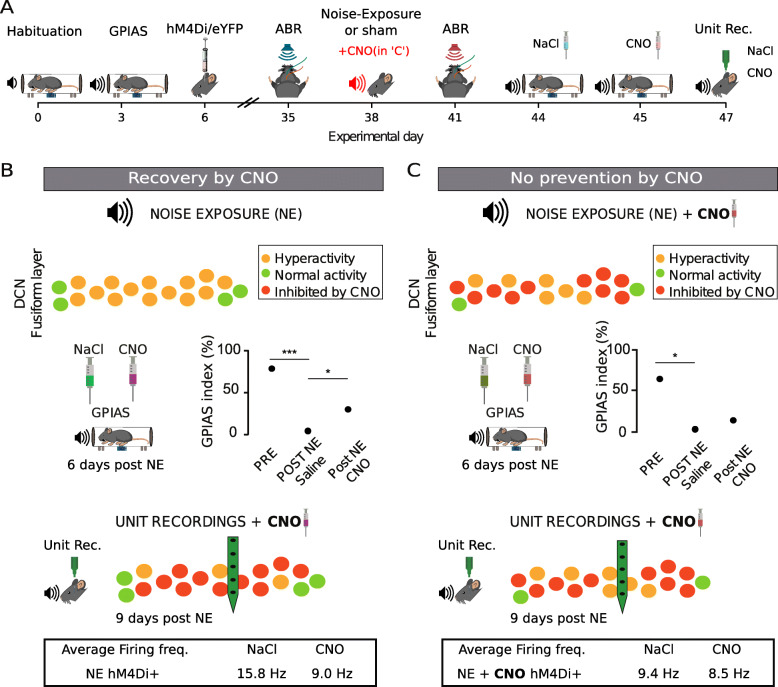
Recovery from tinnitus-behavior when decreasing activity of CaMKII *α*+ DCN neurons acutely but not if activity was chemogenetically reduced during the noise exposure. **A** Schematic outline highlighting the hypothetical difference between experiments of recovery in tinnitus-like animals (**B**) and animals where the aim was to prevent tinnitus (CNO administered before the noise exposure, second set of experiments as shown in **C**, red font). **B** Top, schematic drawing of the DCN fusiform layer showing hypothetical hyperactive neurons following noise exposure. Middle, CNO improved gap detection. Bottom, CNO administration causes a reduction in average firing frequency that may be due to inhibiting hyperactive DCN neurons. **C** Top, schematic drawing showing the DCN fusiform layer chemogenetically inhibited during the noise exposure; however, some neurons are probably not affected by CNO and may still render some neurons hyperactive. Middle, acute CNO exposure does not improve gap detection in mice administered CNO during the noise exposure. Bottom, unit recordings in animals administered CNO during the noise exposure shows that acute additional CNO administration reduces the overall firing frequency less dramatically compared to “B,” suggesting units expressing hM4Di receptors were not hyperactivated during the noise trauma

One interesting finding of our second set of experiments was that, if CaMKII *α*-hM4Di-positive DCN cells still have a role in tinnitus triggering, they are not the only subpopulation involved, since inhibiting them was not enough to prevent GPIAS-confirmed tinnitus. Here, mice still develop tinnitus-like responses in startle suppression tests, but since the CaMKII *α*-hM4Di-positive DCN cells were inhibited during noise exposure, we speculate that no plasticity took place in those cells (Fig. 9). Since mice treated with CNO after noise exposure showed a significant, but yet only partial recovery of GPIAS index, this indicates that not all overactive cells were inhibited by CNO (Fig. 9B) and also explains why mice treated with CNO during noise exposure did not improve the GPIAS index (and were not prevented from developing tinnitus, Fig. 9C). If CaMKII *α*+ units were inhibited during noise exposure, these units would not present long-term potentiation and hyperactivity; therefore, an additional dose of CNO would not counteract any increased firing rate during the GPIAS test. Instead, other neurons (not CaMKII *α*+ cells) would contribute to the GPIAS-confirmed tinnitus seen in this experimental group (Fig. 9A,C).

This study still shows that decreasing activity of overactive CaMKII *α*+ DCN neurons significantly improved acoustic startle suppression, pointing to the importance of restoring inhibition in tinnitus [60]. It has recently been confirmed in vivo that there is extensive feedforward inhibition within the dorsal cochlear nucleus, where one fusiform cell is inhibited by on average three cartwheel cells within an isofrequency layer [61, 62]. Interestingly, the same study show that somatosensory stimuli (pontine nuclei stimulation) decrease feedforward inhibition strength of cartwheel cells and that less inhibitory strength was correlated to increased spontaneous, but not evoked, firing frequency in fusiform cells [61]. Therefore, cartwheel cells can influence spontaneous fusiform activity, but multiple cartwheel cells are needed to regulate sound-driven activity [61]. Interestingly, it has been shown that loud noise exposure can increase vesicular glutamate transporter 2 (VGlut2)-positive puncta in the anteroventral cochlear nucleus, and the granule cell domain, both receiving somatosensory input [20]. Thereby, noise-induced tinnitus appears to generate increased somatosensory drive leading to decreased cartwheel cell inhibition, through long-term depression [63]. However, the decrease in inhibition might be local, as cartwheel cells also inhibit each other on a 1:1 ratio, generating motifs of disinhibition between neighboring cartwheel cells [61]. Hence, the outcome of altered feedforward inhibition and disinhibition depends on local interconnectivity and might contribute to findings that tinnitus frequency varies between animals undergoing the same noise exposure [33, 56].

Recent studies have shown that clozapine-N-oxide cannot cross the blood-brain barrier; instead, CNO reverts to the antipsychotic compound clozapine that crosses the blood-brain barrier and can bind to a variety of neurotransmitter receptors [39]. Still, Manvich et al. [41] showed that the amount of CNO necessary to cause unspecified behavioral changes in mice or rats was 5mg/kg, which is 10 × greater than the dose administered in our study. We observed no systematic change in unit recordings of animals injected with CNO without expressing the hM4Di receptor. Instead, we saw effects specific for inhibiting CaMKII *α*-hM4Di-positive DCN cells, with CNO causing a significant decrease in DCN unit firing rate. Still, some units showed an increase in firing rate. Since hM4Di activity through second messengers leads to membrane hyperpolarization [64], units showing an increase in firing rate after CNO injection are most likely being disinhibited [22]. Still, the effects of CNO on best frequency were not binary (increased or decreased best frequency), as some units did not change best frequency. Interestingly, we found that, in noise-exposed animals, firing rate was not correlated with best frequency, regardless if CaMKII *α*-hM4Di-positive DCN cells were inhibited or not. This is probably due to the analysis of the provided stimulus, as many DCN cells may have best frequencies higher than 16kHz and also respond differently to pure-tones [65, 66]. Hence, some units could have been erroneously classified, for example, as a unit with a low firing rate and broad tuning width, confusing results relating to the effect of CNO. Thereby, no inferences about best frequency and tinnitus-like responses, or the tonotopicity of the DCN, have been made in this study.

Nevertheless, a neuron’s tuning width is an important feature that allows refinement of circuitry responses to stimuli. The literature is still ambiguous on how firing rate and tuning width are correlated in DCN units, since generalized inhibition by intracerebral injection of muscimol (GABAA receptor agonist) was shown to not affect tuning width in DCN units of anesthetized rats [67], while another study showed that bicuculline (a GABA receptor antagonist) increased tuning width while GABA and muscimol decreased tuning width [68]. We found that firing rate was correlated with tuning width, except for animals where CaMKII *α*-hM4Di-positive DCN cells were inhibited during noise exposure. We speculate that forcing CaMKII *α*-hM4Di+ DCN cells to keep a low firing during the noise exposure decoupled firing frequency from tuning width, and thereby only a portion of DCN cells would show hyperactivity due to noise exposure [12, 19]. Interestingly, inhibiting these cells during unit recordings restored the correlation between firing rate and tuning width, perhaps by decreasing activity of cells not affected by the noise exposure, and thereby emphasizing regular activity of the remaining circuitry.

## Conclusions

In summary, our results illustrate the complexity of the DCN circuitry and indicate that decreasing CaMKII *α*-hM4Di-positive DCN cell activity may drastically change DCN circuit electrophysiology. Such changes may underlie improvements related to for example loudness of tinnitus [69] which is of clinical relevance as one neurological treatment effect in tinnitus patients is a decreased loudness and/or decrease of Annoyance index [70, 71]. In conclusion, our results show that CaMKII *α*-hM4Di-positive DCN cells play a significant role in maintaining noise-induced tinnitus in mice, and provide a step towards better understanding the neuronal correlates of noise-induced tinnitus in patients with normal hearing threshold.

## Methods

### Animals

Male C57Bl/6J mice (*n* = 30 + 4 excluded, see GPIAS exclusion criteria) were used at the age of 21 days at first and 2 months at the last experiment and were used for each step of the experimental timeline (see complete timeline in Figs. 1, 2, 3, 4, 5, and 6A). All animal procedures were approved and followed the guidelines of the Ethical Committee of Animal Use (CEUA) from the Federal University of Rio Grande do Norte (CEUA protocol number 051/2015). Animals were housed on a 12-h/12-h day/night cycle and had free access to food and water. All experiments were performed during the light cycle.

### Gap prepulse inhibition of acoustic startle reflex

The gap prepulse inhibition of acoustic startle (GPIAS, [31]) test, based on the acoustic startle reflex in response to sudden loud sounds, was conducted in a sound-shielded room inside a sound-shielded chamber with LED lights. During recordings, the animal was placed inside a clear acrylic tube (Acrilart, Natal, Brasil), dimensions 6.1 ×5.9×5.1cm, with perforated plates closing the tube at both ends. The tube dimensions restricted mice from standing on the back paws. A speaker (Selenium Trio ST400, JBL by Harman, Brazil) was placed 4.5cm away from the restraining tube. In order to measure the animal’s startle reflex, a piezoelectric or a digital accelerometer was mounted to the base plate of the restraining tube. Sound stimulus consisted of blocks of narrow-band uniform white noise at background level, loud intensity (105dBSPL) or silence. Specifically, the stimulus was presented in the following sequence: a random integer value between 12 and 22 s of noise at background level (randomized background noise between trials); 40ms of noise at background level for Startle trials, or 40ms of silence for Gap-startle trials (Gap portion); 100ms of noise at background level (background noise before loud pulse); 50ms of noise at 105dBSPL (loud pulse); and 510ms of noise at background level (final background noise). Timestamp marks were used only for the loud pulse. The bands of frequencies tested were 8–10, 9–11, 10–12, 12–14, 14–16, and 8–18kHz. Background noise level was, for the initial GPIAS test, 60dBSPL. For GPIAS after noise exposure, background noise level was adjusted to 10dBSPL above the hearing threshold for the frequency tested.

Before each session, the acrylic tube was cleaned with ethanol (70%) and next with water to remove residual smell of ethanol. Animals were habituated by handling for 10 min in the test room for two consecutive days followed by 3 days of acclimatization where animals were placed in the GPIAS tube and exposed to background noise, and next returned to their homecage. A successful acclimatization and habituation was considered when animals enter freely and do not urinate or defecate in the tube. After the habituation/acclimatization period, animals were screened for gap detection capability. The animals were placed in the restraining tube and left in the recording chamber for 5 min, allowing the animal to stay calm and stop exploring the chamber [72]. The test consisted of 18 trials per band of frequency tested, 9 with gap (Gap-startle trials) and 9 with noise filling the gap portion of the stimulus (Startle trials), presented pseudo-randomly. The GPIAS sessions were carried out at 3 time points for each animal: initially, for screening animals before being included in experimental groups (see analysis for exclusion criteria), then in the end of the experiment timeline in the following NaCl injections, and the following day 30 min after CNO (0.5mg/kg, dissolved in dimethyl sulfoxide (DMSO) at 3.3mg/ml, then diluted in NaCl to the final concentration of 50 *μ*g/ml) administration. Each GPIAS session lasted between 23 and 41 min in total (depending on the randomization of interpulse intervals). Upon the end of the session, animals were returned to their home cage.

### Virus injection

Mice were anesthetized with an i.p. injection of ketamine-xylazine combination at 90/6 mg/kg. When necessary, additional ketamine at 45 mg/kg was applied during surgery. The mouse was next mounted into a stereotaxic device resting on a heating block (37^∘^C). The eyes were covered with dexpanthenol to prevent ocular dryness and povidone-iodine 10% was applied onto the skin of the animal’s head to avoid infections. The skin was anesthetized with lidocaine hydrochloride 3% before a straight incision was made, and hydrogen peroxide 3% was applied onto the exposed skull to remove connective tissue and visualize bone sutures. A small hole was carefully drilled at bilateral DCN coordinates (anteroposterior; AP = −6.24mm and mediolateral; ML = ±2.3mm) using a dental microdrill. Next aliquoted virus (experimental: rAAV5/CaMKII *α*-HA-hM4D(Gi)-IRES-mCitrine, UNC Vector Core #AV4617C, viral concentration of 1.6 ×10^12^vm/ml; or control: rAAV5/CaMKII *α*-eYFP, UNC GTC Vector Core #AV4808D, 4.4 ×10^12^vm/ml) was rapidly thawed and withdrawn (1.5 *μ*l) using a syringe pump (Chemyx NanoJet infusion pump). The needle (10 *μ*l Nanofil syringe with a 34-gauge removable needle) was slowly inserted into the brain (dorsoventral; DV = -4.3mm) and 0.75 *μ*l of virus was infused (0.15 *μ*l/min). At completed infusion, the needle was kept in the DV coordinate for 5 min to allow for the virus to diffuse and then the needle tip was retracted to -3.8mm DV, where 0.75 *μ*l of virus was again infused at the same rate. After the second infusion, the needle was kept in place for 10 min, to allow for a complete diffusion into the target area, before carefully removed. The same procedure was performed bilaterally. Following injections, the skin was sutured, lidocaine hydrochloride 3% applied over the suture, and 200 *μ*l of NaCl subdermally injected for rehydration. Animals were monitored until fully recovered from anesthesia.

### Auditory brainstem responses

Similarly to the GPIAS setup, the speaker was connected to a sound amplifier connected to a sound card and placed 4.5cm away from a stereotaxic frame. Field potentials (auditory brainstem responses (ABRs)) were recorded using two chlorinated coiled Ag/AgCl electrodes as a recording and a reference electrode (1k *Ω* impedance). The electrodes were connected to the RHD2132 headstage through a DIP18-Omnetics connector, connected to Open-ephys board. Animals were anesthetized with an i.p. injection of ketamine-xylazine combination at 90/6 mg/kg and fitted to the stereotaxic frame, placed on an electric thermal pad and kept at 37^∘^C. Dexpanthenol or NaCl was applied on the animal’s eyes to avoid drying of the ocular surface. Next, the scalp was disinfected with polividone-iodine (10%) and two small incisions were made: one in the skin covering the lambda region and another in the skin over the bregma region. The electrodes were placed subdermally into the incisions and the ground was connected to the system ground. The electrode at bregma was used as reference, and the electrode over lambda was used for recording. Sound stimuli consisted of narrow-band uniform white noise pulses (3ms), presented at 10Hz for 529 repetitions for each frequency and intensity tested. The frequency bands tested were the same used for GPIAS: 8–10, 9–11, 10–12, 12–14, and 14–16kHz (with exception for the 8–18kHz frequency band), and sound pulses were presented at decreasing intensities from 80 to 35dBSPL, in 5dBSPL steps, with 10s of silence between different intensities. After the test, electrodes were removed, lidocaine hydrochloride 3% was applied on the incisions, and 200 *μ*l of NaCl was injected subdermally for rehydration. Animals were monitored after surgery until fully recovered from anesthesia and then returned to their home cage.

### Noise exposure

Anesthetized mice were placed inside a sound-shielded chamber, inside an acrylic tube, in an acoustically shielded room, with a speaker placed 4.5cm in front of the head of the mouse. Noise exposure consisted of narrow-band uniform white noise presented at 90dBSPL, 9–11kHz, for 1 h. The animal was left in the acrylic tube, in the sound-shielded chamber for 2h following noise exposure, since external noise before and/or following noise exposure can interfere in tinnitus development [73–75]. During noise exposure and the silence period, the animal was monitored each 15 min and later returned to its homecage. Animals were given 2 days to recover before any further procedures. In some experiments, CNO (0.5mg/kg) was given 30 min prior to noise exposure.

### In vivo unit recording

Animals were anesthetized with an i.p. injection of ketamine-xylazine combination at 90/6 mg/kg and placed into the stereotaxic frame similar to for ABR recordings. A small craniotomy was drilled above the left DCN (AP = −6.24mm ML = −2.3mm) and a silicon depth probe (16 channels, 25 or 50 *μ*m channel spacing, 177 *μ*m recording site area, 5mm long shank; NeuroNexus A16) dipped in fluorescent dye (1,1 ^′^-dioctadecyl-3,3,3 ^′^,3 ^′^-tetramethylindocarbocyanine perchlorate; DiI, Invitrogen) for 10 min (for probe position) before lowered into the DCN (DV = -4.3mm). A coiled Ag/AgCl wire soldered to a jumper wire was used as reference. The probe and reference wire were both connected to a headstage (RHD2132) through an adaptor (DIP18-Omnetics) connected to the Open-ephys board, recording at a sampling rate of 30kHz. Sound stimulus consisted of narrow-band uniform white noise pulses (3ms) as described for ABRs, presented at 10Hz for 529 repetitions for each frequency and intensity tested. Spontaneous activity was recorded for 5 min, then the animal received an i.p. injection of NaCl, then sound stimulation started 30 min later. Subsequently, the same procedure was repeated for CNO (0.5mg/kg). At the end of the recording session, the animals were either sacrificed by intracardial perfusion (20mL PBS and 20mL paraformaldehyde 4%) or by an overdose of ketamine followed by decapitation.

### Data analysis

All scripts used for controlling devices, stimulation control, and data analysis are available online (LabScripts git repository, [76]). The operating system of choice was Gentoo GNU/Linux, due to its flexible management of libraries [77]. Recordings were done using Open-ephys GUI [78]. Microcontrollers and sound cards were controlled using SciScripts [79], and the sounddevice python library [80] was used to read and write signals from/to the sound card. Calculations were done using Scipy [81], Numpy [82], and SciScripts [79], and all plots were produced using Matplotlib [83]. Spikes were detected and clustered using SpyKING Circus [84], and visual inspection was performed using Phy [85].

GPIAS signal was bandpass filtered from 70 to 400Hz for piezoelectric recordings and lowpass filtered below 50Hz for accelerometer recordings. Data was sliced 200ms around the loud pulse onset. For accelerometer recordings, the absolute values of the three axes were averaged. The 9 Gap-startle trials of the same frequency band were averaged, as were the 9 Startle trials. The instantaneous amplitude of the signal was calculated as the magnitude of the analytic representation of the averaged signal using the Hilbert transform. The amplitude of the response was defined as the mean instantaneous amplitude of the 100-ms range after the loud sound pulse subtracted by the mean instantaneous amplitude from −100 to 0ms before the loud pulse, which corrects for baseline offsets. The GPIAS index was calculated as

$$\left(1-\left(\frac{GapStartle}{Startle}\right)\right)*100 $$

where *Startle* is the amplitude of response to Startle trials and *Gapstartle* is the amplitude of response to Gap-startle trials. The most affected frequency for each animal was calculated as the frequency with the greatest index shift from before to after noise exposure. On screening GPIAS capability before including animals into the study, animals that did not show a startle suppression of at least 30% [13] in Gap-startle vs Startle trials for all frequencies were re-tested on the next day only on failed frequencies. Animals that still did not show a startle suppression by the silent gap of at least 30% for at least two frequencies were excluded from further experiments. Group data is shown as boxplots, where horizontal lines show the median, triangles show mean, circles show outliers, and whiskers bounding 99% of the data points. Effects of noise exposure, treatment, and frequency were evaluated using two-way repeated measures ANOVA (Noise exposure × Frequency and Treatment × Frequency), and pairwise comparisons were performed using two-tailed paired Student’s *t*-test, Bonferroni-corrected for the number of frequency bands tested. Comparisons between the most affected frequencies at different sessions were done using one-way repeated measures ANOVA (noise exposure and treatment). Assuming a medium effect size, with *n* = 11, 7, 6, and 6 (noise-exposed hM4Di+, noise-exposed eYFP+, noise-exposed +CNO hM4Di+ and noise-exposed +CNO eYFP+ groups, respectively), the ANOVA statistical power was of 94.5%, 81.3%, 76.5%, and 76.5%, respectively.

ABR recordings were filtered using a 4th order butterworth digital bandpass filter (600–1500Hz), and data was sliced 3ms before to 9ms after each sound pulse onset and the 529 trials were averaged. ABR peaks were detected in the highest intensity response as values one standard deviation (SD) above the mean, larger than the previous value, and larger or equal to the next value. Next, each decreasing intensity was screened for peaks where a “valid peak” follows the above criteria, is 1SD above its mean, and, in addition, has to be preceded by a peak in the previous intensity, displaying an increased latency compared to the peak in the higher intensity response. Hearing threshold was defined as the lowest sound intensity where a peak can be detected following the above criteria. If the threshold is defined as 35dBSPL, the animal’s hearing threshold is considered as ≤35dBSPL. As for GPIAS results, group data is shown as boxplots, where horizontal lines show the median, triangles show mean, circles show outliers, and whiskers bounding 99% of the data points. Data is reported as mean ± standard error of the mean (SEM). Due to the non-normal distribution of the ABR thresholds (evaluated using the Shapiro test), differences were evaluated using the Kruskal-Wallis test (noise exposure and frequency) followed by pairwise comparisons using the Wilcoxon test, Bonferroni-corrected for the number of frequency bands tested. Differences in ABR wave 1 amplitudes at 80dBSPL and growth functions were evaluated using two-way repeated measures ANOVA (Noise exposure × Frequency) and three-way repeated measures ANOVA (Noise exposure × Frequency × Intensity), respectively. The calculated ANOVA statistical power, for *n* = 11, 7, 6, and 6, was 98.7%, 88.2%, 80.8%, and 80.8% for the noise-exposed hM4Di+, noise-exposed eYFP+, noise-exposed +CNO hM4Di+, and noise-exposed +CNO eYFP+ groups, respectively.

Spikes from unit recordings were detected and clustered using the following parameters: 4th order butterworth digital bandpass filter from 500 to 14250Hz; detect negative spikes; single threshold from 2∼4.5× SD; 3 features per channel. Peri-stimulus time histograms (PSTHs) were calculated by summing occurrence of spikes in a time window of 100ms around each TTL (50ms before and 50ms after the TTL) and presented as number of spikes per time, where each bin corresponds to 1ms. Units were classified as responding units as described by Parras et al. [86]. Spike rate was calculated as spike events per second along all the recordings (including the stimulation period). The firing rate of each unit was calculated for each frequency and intensity tested, and plotted as frequency-intensity-firing rate pseudocolor rectangular grid plots, then firing rate was bilinearly interpolated, upsampling 3 × in frequency and intensity dimensions. Unit tuning width was calculated as the mean of the normalized firing rate for each frequency tested at 80dBSPL; therefore, higher values represent broader tuning curves. Unit best frequency was defined as the sound frequency that elicited the highest firing rate. Group data is reported as mean ± SEM, and paired two-tailed Student’s *t*-test with unequal variance was applied to compare firing rate between neurons. Assuming a medium effect size, with *n* = 122, 102, 85, and 91 units (noise-exposed hM4Di+, noise-exposed eYFP+, noise-exposed +CNO hM4Di+, and noise-exposed +CNO eYFP+ groups, respectively), the *t*-test statistical power was >99% for all four groups. Correlation between unit features (firing rate, tuning width, and best frequency) was calculated as Pearson correlation coefficient and *p*-value for testing non-correlation. The reported *p*-values were Bonferroni-corrected when the same dataset was used for multiple comparisons.

## Supplementary Information

**Additional file 1** **Fig. S1.** Bimodal unit responses seen upon CNO administration in hM4Di+ mice. A) Left; Firing rate in response to 80dBSPL at best frequency for all units (n = 122) from hM4Di+ mice in response to NaCl or CNO. Middle; Only units decreasing (n = 96) firing rate upon CNO administration. Right; Units increasing (n = 26) firing rate after CNO administration. B) Same as ‘A’ but for Tuning width, with units decreasing (n = 71) and increasing (n = 51) tuning with after CNO administration. C) Same as ‘A’ for representation of Best frequency in kHz, with units decreasing (n = 30), increasing (n = 56) or maintaining (n = 36) Best frequency response upon CNO administration. Note that units responding to sound do not need to be CaMKII *α*+, the unit altered firing properties are in response to sound when CNO is decreasing activity of CaMKII *α*+ units of the DCN circuit. *: p <0.05; ***: p = 1.3e-04.

**Additional file 2** **Fig. S2.** Bimodal unit responses seen upon CNO administration in hM4Di+ animals that were treated with CNO also during noise exposure. A) Left; Firing rate in response to 80dBSPL at best frequency for all units (n = 85) from hM4Di+ mice in response to NaCl or CNO. Middle; Only units decreasing (n = 54) firing rate upon CNO administration. Right; Units increasing (n = 31) firing rate after CNO administration. B) Same as ‘A’ but for Tuning width, with units decreasing (n = 31) and increasing (n = 54) tuning with after CNO administration. C) Same as ‘A’ for representation of Best frequency in kHz, with units decreasing (n = 39), increasing (n = 26) or maintaining (n = 20) Best frequency response upon CNO administration. Note that units responding to sound do not need to be CaMKII *α*+, the unit altered firing properties are in response to sound when CNO is decreasing activity of CaMKII *α*+ units of the DCN circuit. *: p <0.05.

## Data Availability

All data generated or analyzed during this study are included in this published article and supplementary information files.

## Abbreviations

ABR: auditory brainstem response
AP: anteroposterior
CaMKII *α*: Ca^2+^/Calmoduline kinase 2 *α*
CEUA: Ethical Committee in Use of Animals
CNO: clozapine-N-oxide
dBSPL: decibels sound pressure level
DCN: dorsal cochlear nucleus
DV: dorsoventral
GPIAS: gap prepulse inhibition of acoustic startle
hM4Di: human M4 Designer Receptors Exclusively Activated by Designer Drugs
HFS: high-frequency stimulation
i.p.: intraperitoneal
ML: mediolateral
PSD: power spectrum density
PSTH: per-stimulus time histogram
RMS: root mean square
UFRN: Federal University of Rio Grande do Norte
VGlut: vesicular glutamate transporter
