# Supplementary figures and images for: Decreasing dorsal cochlear nucleus activity ameliorates noise-induced tinnitus perception in mice
Source: BMC Biol. 2022 May 12;20:102. doi: 10.1186/s12915-022-01288-1 (PMC9097071; doi:10.1186/s12915-022-01288-1)

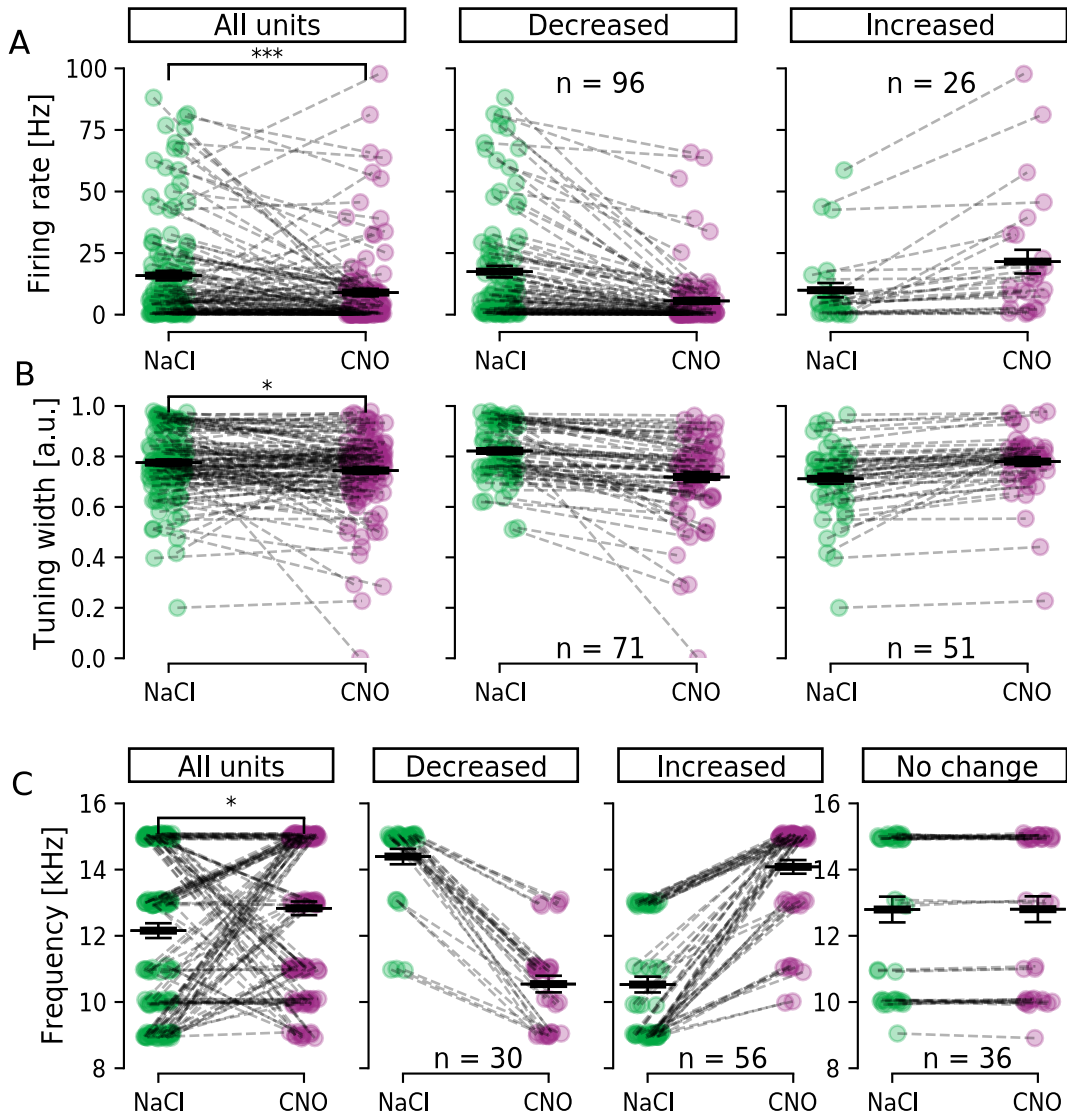

Supplement: Supplementary file 1 — Additional file 1 Fig. S1. Bimodal unit responses seen upon CNO administration in hM4Di+ mice. A) Left; Firing rate in response to 80dBSPL at best frequency for all units (n = 122) from hM4Di+ mice in response to NaCl or CNO. Middle; Only units decreasing (n = 96) firing rate upon CNO administration. Right; Units increasing (n = 26) firing rate after CNO administration. B) Same as ‘A’ but for Tuning width, with units decreasing (n = 71) and increasing (n = 51) tuning with after CNO administration. C) Same as ‘A’ for representation of Best frequency in kHz, with units decreasing (n = 30), increasing (n = 56) or maintaining (n = 36) Best frequency response upon CNO administration. Note that units responding to sound do not need to be CaMKII α+, the unit altered firing properties are in response to sound when CNO is decreasing activity of CaMKII α+ units of the DCN circuit. *: p <0.05; ***: p = 1.3e-04. [file 12915_2022_1288_MOESM1_ESM.pdf]

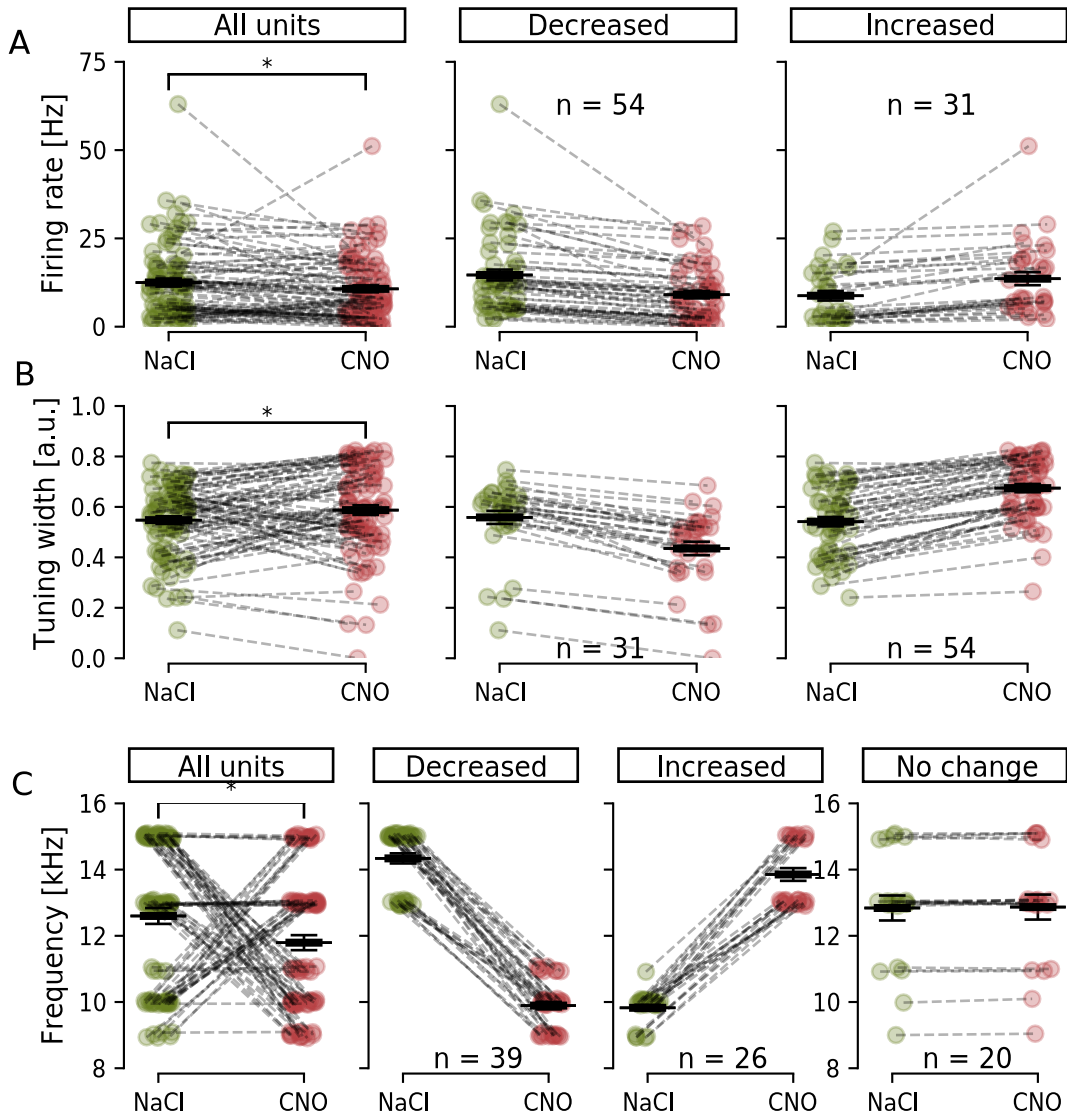

Supplement: Supplementary file 2 — Additional file 2 Fig. S2. Bimodal unit responses seen upon CNO administration in hM4Di+ animals that were treated with CNO also during noise exposure. A) Left; Firing rate in response to 80dBSPL at best frequency for all units (n = 85) from hM4Di+ mice in response to NaCl or CNO. Middle; Only units decreasing (n = 54) firing rate upon CNO administration. Right; Units increasing (n = 31) firing rate after CNO administration. B) Same as ‘A’ but for Tuning width, with units decreasing (n = 31) and increasing (n = 54) tuning with after CNO administration. C) Same as ‘A’ for representation of Best frequency in kHz, with units decreasing (n = 39), increasing (n = 26) or maintaining (n = 20) Best frequency response upon CNO administration. Note that units responding to sound do not need to be CaMKII α+, the unit altered firing properties are in response to sound when CNO is decreasing activity of CaMKII α+ units of the DCN circuit. *: p <0.05. [file 12915_2022_1288_MOESM2_ESM.pdf]
